# Perspectives on Optimized Transcranial Electrical Stimulation Based on Spatial Electric Field Modeling in Humans

**DOI:** 10.3390/jcm13113084

**Published:** 2024-05-24

**Authors:** Jose Gomez-Tames, Mariano Fernández-Corazza

**Affiliations:** 1 Department of Medical Engineering, Graduate School of Engineering, Chiba University, Chiba 263-8522, Japan; 2 Center for Frontier Medical Engineering, Chiba University, Chiba 263-8522, Japan; 3 LEICI Institute of Research in Electronics, Control and Signal Processing, National University of La Plata, La Plata 1900, Argentina

**Keywords:** tES, tDCS, tACS, FEM, transcranial electrical stimulation, electric field, current density, neurostimulation, optimization, brain template, computational model

## Abstract

**Background**: Transcranial electrical stimulation (tES) generates an electric field (or current density) in the brain through surface electrodes attached to the scalp. Clinical significance has been demonstrated, although with moderate and heterogeneous results partly due to a lack of control of the delivered electric currents. In the last decade, computational electric field analysis has allowed the estimation and optimization of the electric field using accurate anatomical head models. This review examines recent tES computational studies, providing a comprehensive background on the technical aspects of adopting computational electric field analysis as a standardized procedure in medical applications. **Methods**: Specific search strategies were designed to retrieve papers from the Web of Science database. The papers were initially screened based on the soundness of the title and abstract and then on their full contents, resulting in a total of 57 studies. **Results**: Recent trends were identified in individual- and population-level analysis of the electric field, including head models from non-neurotypical individuals. Advanced optimization techniques that allow a high degree of control with the required focality and direction of the electric field were also summarized. There is also growing evidence of a correlation between the computationally estimated electric field and the observed responses in real experiments. **Conclusions**: Computational pipelines and optimization algorithms have reached a degree of maturity that provides a rationale to improve tES experimental design and a posteriori analysis of the responses for supporting clinical studies.

## 1. Introduction

Transcranial electrical stimulation (tES) refers to several non-invasive neuromodulation techniques that generate an electric current in the brain using two or more surface electrodes attached to the scalp [1,2]. tES has demonstrated applications in various clinical trials (e.g., [3,4]). However, clinical results have been moderate and heterogeneous (e.g., [5]). One reason is the variability in delivered intracranial currents among different patients for the same stimulation parameters and lack of control of the delivered electric currents (e.g., [6,7]). Therefore, it is difficult to infer the involvement of a specific brain area in the examined clinical or behavioral outcomes. Thus, it is important to understand how the current is distributed in the brain during tES. Computational modeling estimates intracranial electric current distribution and intensity, enabling users to regulate current for specific brain areas or networks within and across subjects to guide montage selection. Developing optimization algorithms for montage selection can also allow better control over the electric field’s intensity and direction.

Analytical solutions of the spatial distribution of the current flowing in the brain from surface electrodes were presented in the last century using concentric spheres representing the scalp, skull, and brain [8,9] and CSF [10]. From the 2000s, more realistic electrode montages were initially considered based on numerical simulation using a spherical model [11,12] and low-resolution head models based on MRI data [13,14]. The model progressed towards high-resolution descriptions of the head tissues, such as gyrification (e.g., [15]).

In the last ten years, significant progress has been made in generating individualized human head models from medical images in systematic manner (pipelines) that account for personalized anatomical features [16,17,18]. The analysis of the individualized electric field clarified the role of stimulation parameters and anatomical aspects shaping the electric field in the brain cortex [19,20] and deep brain areas [21,22]. Ongoing efforts aim to clarify the relationship between tES-generated electric fields and physiological/behavioral responses [23]. Recently, not only has individualized-level knowledge of the electric field been gained, but population-level knowledge of the electric field distribution has also gained interest based on registration techniques of the individualized electric field into brain templates [24,25,26,27]. In addition to these developments, tES optimization has been increasingly investigated, leading to different computational algorithms (e.g., [28,29,30]). These algorithms find the optimal electric currents to inject through each electrode or the optimal electrode positions, given a head model, a target to stimulate, and safety and hardware constraints. Also, there are different optimization criteria, for instance, maximizing the directional component of the electric field at the target, maximizing the module of the electric field, or maximizing the focality, among others. Moreover, some of these algorithms were adapted to optimize a modulated tES technique [31] (known as temporal transcranial interferential stimulation or tTIS [32]). Most of these tES optimization methods have been tested using computational simulations, with only a few studies using them in real experiments.

Reviews on tES electric field analysis have been published on computational studies informing the montage design and interpretation of the results [33] and on developed pipelines and toolboxes for individualized electric field analysis [34]. More recently, a mini review presented retrospective and prospective applications of electric field analysis [23]. The present review gives a new focus by deepening individualized-level and population-level knowledge of the electric field computation together with optimization techniques that maximize the electric field on the desired target(s) in the human brain. This review is intended for research groups working on research and clinical applications interested in adopting computational models with state-of-the-art electric field analysis.

## 2. Outline of tES Computational Model for Electric Field Analysis

**Figure 1:**
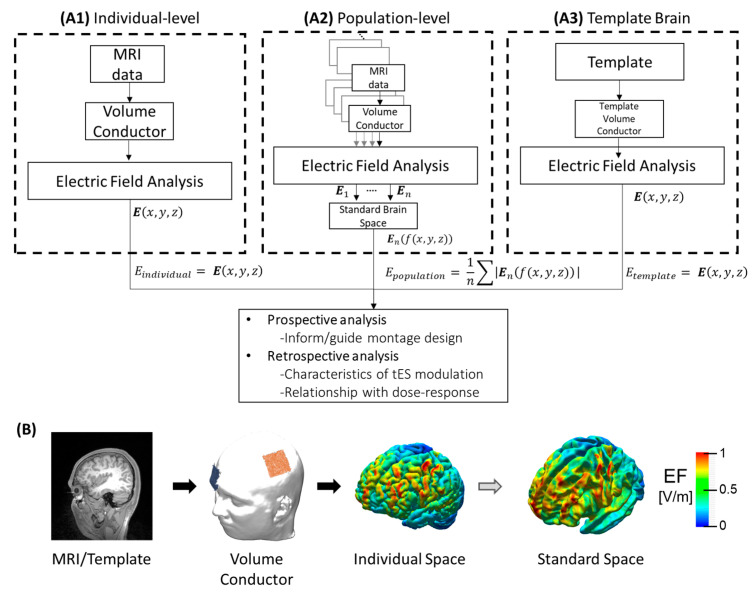
Summary of computational analysis approaches for tES analysis. (**A1**) Electric field $\vec{E}(x,y,z)$ obtained from individual MRI data. (**A2**) Population-level which corresponds to statistical analysis (e.g., mean) of the registered electric fields $\vec{E}(f(x,y,z))$ from different individuals in the standard space where *f:*ℝ*^3^*→ℝ*^3^* is the function assigning the spatial position of the electric field in the individual space to standard space. (**A3**) The electric field is estimated from a template model, which could be an averaged MRI of a target population. (**B**) Illustration of electric field estimation on the brain cortex, from left to right: MRI of the individual; volume conductor after segmentation, meshing, assigning electric properties of each tissue, and adding the electrode montage; electric field distribution on the brain cortex in the individual space; and registration of the individual electric field into a template in the standard space.

The tES-generated electric field is computed using a realistic human head model representing the tissue-dependent conductivity distribution (volume conductor model or current flow models based on individual-based or template-based head models). Electric field analysis is illustrated in Figure 1. This section presents the technical aspects of electric field analysis for tES.

### 2.1. tES Parameter Space

In tES, electric currents or voltages are applied to the head following some injection pattern using at least two transcranial electrodes [1,2]. The current injection pattern refers to a list of electric current levels applied to the head at each electrode. The stimulation parameter space includes injection current intensity and duration, polarity, frequency, and phase (depending on the type of tES protocol). Conventional tDCS protocols are established within a range of intensities and durations (≤30 min, ≤2 mA, ≤7.2 C) for safety and also adopted in tACS studies [35,36]. However, parameter selection is vast and yet to be fully explored. Various studies have expanded parameter space by extending the range of current intensity with currents up to 4 mA [37,38].

The electrode design and placement are also part of the stimulation parameter space. The electrodes are usually made of metal or rubber covered by a soaked sponge or a conductive medium in contact with the scalp [20,39]. The same electrodes used to record electroencephalography (EEG) have been used for current injection in tES experiments (e.g., [40]).

These stimuli parameters control different spatiotemporal characteristics of the generated electric field in the brain. In the tES electric field modeling and tES optimization literature, the spatial and temporal characteristics have typically been decoupled based on the quasi-static approximation of Maxwell’s equations [41]. In particular, the decoupled spatial distribution and intensity of the electric field are determined by the electrodes (material, size, design, position, number) and by the anatomical and electrical properties of the human head tissues and fluids. The use of multiple electrodes, such as high-density and multifocal montages, together with the current injection pattern can control the electric field shape with higher focality [12,28]. The temporal stimulation waveform will change the scale of the spatial map according to the instantaneous intensity of the applied waveform because scaling the current injection pattern has a linear relationship with the resulting electric field intensity in the brain. Thus, considering only the spatial information of the electric field (independent of the temporal waveform) has been convenient and fundamental to estimating the intensities of the electric fields in the different brain regions produced by different montages and to determining convenient or optimal montages to enhance the electric field intensity, focality or directionality at the desired targets. In the case of tTIS, the temporal pattern depends on the spatial alignment of the two interfering fields and, thus, the spatial and temporal aspects of the stimulation are more closely correlated. However, the modulation depth of tTIS can be depicted as a purely spatial map [32,42]. The details of the electric field calculation are presented in the next subsection.

Note that this review covers studies on the tES-generated electric field spatial distribution. The specific dynamics of the electric field interaction require the application of neural modulation models driven by spatiotemporal electric fields (see future directions in Section 4.3). It is also important to keep in mind that as the frequency is closer to 10 kHz [43], the quasi-static assumption becomes weaker, and the spatial characteristics of the electric field are dependent on the injection current waveform.

### 2.2. Physics of tES

The external electric currents or voltages applied to the head produce an internal electric field or current density distribution in the whole head, shaped by the electrical and geometrical properties of the tissues. This electric field distribution can be computed on virtual head models to estimate the electric fields in the brain. This problem of computing the resulting electric field on a given head model and for a given current injection pattern is known as the tES forward problem (FP). The FP is a classic electromagnetic problem with boundary conditions governed by the physical laws of electromagnetism: Maxwell’s equations [44]. For low frequencies (less than ~10 kHz), the quasistatic approximation of Maxwell’s equation is accepted because the wavelength is much larger than the human head dimensions at these frequencies. The boundary conditions can be either Dirichlet, if known voltages are applied, Neumann, if known currents are applied, or a combination of both, but typically, electric currents (Neumann conditions) are applied.

From the quasistatic Maxwell–Faraday equation $\vec{\nabla }\times \vec{E}=0$, the electric field is related to the electric potential Φ by $\vec{E}=-\vec{\nabla }\Phi$. Then, the current density is $\vec{J}=-\overset{⃡}{\sigma }\cdot \vec{\nabla }\Phi$. The divergence operator accounts for the sources and sinks (or expansions and contractions) of an electric field. In tES, the only generators are the boundary conditions, and thus, inside the solid (Ω) it is assumed that there are no field generators resulting in $\vec{\nabla }\cdot \vec{J}=0$ in Ω. Note that the internal brain activity is neglected in tES assuming that the imposed electric field is much larger than the intrinsic electric fields generated by the neuronal activity. Regarding the boundary conditions, there are two options: the electrodes can be modeled as points or as a surface with a contact impedance (also known as the complete electrode model) [45]. Finally, the FP with Neumann conditions and the electrode boundary condition can be mathematically stated as follows:

(1) $$\left\{\begin{array}{ll} \vec{\nabla }\cdot \left(\overset{⃡}{\sigma }\cdot \left(\vec{\nabla }\Phi \right)\right)=0 & \mathrm{in}\;\Omega \\ \left(\overset{⃡}{\sigma }\cdot \left(\vec{\nabla }\Phi \right)\right)\cdot \hat{n}=0 & \mathrm{in}\;\delta \Omega \\ \Phi +z_{l}\left(\left(\overset{⃡}{\sigma }\cdot \left(\vec{\nabla }\Phi \right)\right)\cdot \hat{n}\right)=V_{l} & \mathrm{in}\;E_{l}\\ \int_{E_{l}}\left(\overset{⃡}{\sigma }\cdot \left(\vec{\nabla }\Phi \right)\right)\cdot \hat{n}\;dS=I_{l} & \mathrm{in}\;E_{l} \end{array}\right.\;\;\;,$$

where $\overset{⃡}{\sigma }$ is the conductivity tensor, δΩ is the solid boundary, $\hat{n}$ is normal to the boundary vector, $I_{l}$ is the injected current at electrode $E_{l}$, $V_{l}$ is the electric potential at electrode $E_{l}$, and $z_{l}$ is the electrode $E_{l}$ contact impedance. The first equation is the Poisson equation, stating that there are no current density generators inside the head; the second equation states that the current density cannot enter or leave the scalp in the regions where there are no electrodes; the third equation means that the electrode potential is the surface potential plus the drop due to the contact impedance; and the fourth equation establishes that the normal component of the current density in the electrode contact surface equals the injected current.

### 2.3. TES Forward Problem Computation

The FP stated in Equation (1) has an analytic solution involving infinite series when the geometries are regular (e.g., spheres or cubes) [46,47,48]. For the geometrically realistic models that are used nowadays, numerical solutions are required. These numerical solutions solve the differential equations, such as Equation (1), by parcellating or meshing the domain or the surfaces into simple elements (typically triangles, tetrahedrons, or prisms) and assuming simpler order piecewise functions in each element. The more elements the mesh has and the larger the order of the functions is, the more accurate the solutions are. The typical methods are the boundary element method (BEM) [49], the finite element method (FEM) [50], and the finite difference method (FDM) [51]. In all cases, the problem of Equation (1) is converted into a linear system of equations Ku=f, where the stiffness matrix K accounts for the geometry and electrical properties of the tissues, f stands for the electromagnetic sources, and u is the unknown electric potential at the mesh nodes and electrodes.

The FEM method is widely used, and it is based on dividing the whole volume into elementary units (typically tetrahedrons or cubes). The meshing process depends on the element type. For cubes or hexahedrons, the meshing can be simpler and derived directly from the MRI voxel space. The tetrahedral meshes are more complex to build but have the advantage that they can be more flexible, allowing for smooth surfaces [52,53].

### 2.4. Individualized Head Model

**Figure 2:**
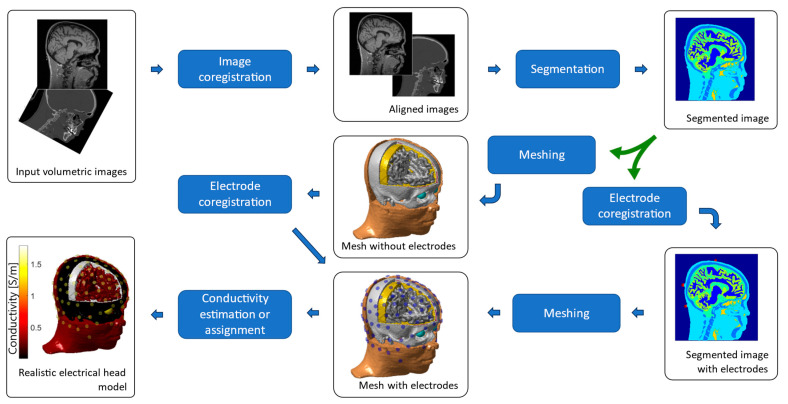
Generation of individual head models based on finite elements. The input images are aligned and segmented. The segmented image can be meshed to obtain the tissue surfaces, or the electrodes can be aligned to the segmented image and added to the segmentation before the meshing process takes place. The final mesh is typically composed of tetrahedral or hexahedral elements, filling the whole head volume. The electrical head model is completed by assigning electrical conductivity values to each tissue, either isotropic or anisotropic and homogeneous or inhomogeneous.

The construction of a geometrically realistic head model, either individual or atlas-based, is key to the accuracy of the tES simulation. Figure 2 shows a diagram of the typical workflow. The first step is the segmentation of a structural T1 magnetic resonance (MR) image, either alone or combined with a T2 or CT image, into the different tissues of the head. A T1 image allows good differentiation of various tissue boundaries, although it is difficult to distinguish the interface between the skull and CSF. In a T2 image, the CSF appears bright, so it can be used to distinguish it from the skull. Another possibility is to use a combination of T1 with a CT image. CT imaging allows the skull to be separated from the rest of the tissues with great precision, allowing the construction of well-detailed models of the skull. If two imaging modalities are used, it is necessary to perform an alignment and resampling or coregistration between both images.

From the coregistered images, the segmentation consists of labeling each voxel with a tissue. Automatic segmentation of the brain and non-brain tissues from MRI data can be obtained using different image analysis software, such as FreeSurfer [54], Statistical Parametric Mapping (SPM), or FMRIB software library (FSL) [55], which can be sped up using a deep neural network [56,57]. These different image analysis tools have been incorporated into different head model generation and electric field calculation pipelines, such as ROAST [17] and SimNIBS [16]. The segmented number of tissues varies from simpler head models of three or four tissues (brain, skull, scalp, and eventually CSF) (e.g., [58]) to more complex head models up to, for instance, 18 tissues, including major tissues such as white matter, gray matter, eyeballs, soft bone or compact bone and details such as dura, blood vessels, fat, basal ganglia or internal air [59]. The segmentation, meshing, and forward problem-solving processes can also be performed using commercial tools.

From the segmented image, the next step includes surface meshing (as required for BEM) and volumetric mesh (as required for FEM). For BEM, the Tetgen algorithm allows surface meshing to be performed from the segmented volumetric image, although it requires that the surfaces do not intersect. For FEM, there are at least two alternatives: the first is to generate the tetrahedral mesh from the surfaces used in BEM, and the second is to construct the mesh directly from the segmented image. The first one allows building meshes with better quality elements, although all surfaces must be closed and concentric (resulting in layers without holes). The second one allows complex surfaces to be generated, including holes or isolated areas. However, some surfaces may have small “spikes” or sharp points that result in numerical errors. It is also possible to use a combination of both methods, i.e., use the second alternative for complex surfaces such as the skull and scalp and use meshing from surfaces for internal layers such as CSF, gray matter, and white matter, which are concentric and closed (as performed, for instance, in [59]).

The coregistration of the model and the electrodes can be carried out either before the segmentation step or at the end after the meshing step. It is possible to obtain the electrode coordinates using photographic images [60] or magnetic sensors [61]. For more details on the electrode model implementation, please refer to [20,62]. More advanced models include white matter anisotropy from diffusion tensor imaging (DTI), major blood vessels, or the dura mater. More details are found in a review that compares different pipelines for tES computational modeling [34].

### 2.5. Brain Template (Standard Space)

The human brain’s structure is variable in size, curvature, and functional topology. Variation occurs not only across individuals but also across the brain lifespan. This substantial intra- and inter-variability hinders spatial comparison, requiring methods for determining population-level knowledge of electric field distributions. Templates have been created by averaging anatomical images from a large sample of subjects. They can be either volumetric-based or surface-based templates. Registration is the process of transforming an image into the coordinate space of another [54,63], such as in the FreeSurfer tool [64], Flirt in FSL [54] or Spherical Demons [65].

Various templates have been implemented. The MNI305 was the first MNI template corresponding to 305 T1-weighted MRI brains (mean age = 23.4 ± 4.1 years). First, 250 MRI images were scaled to match the manually defined landmarks to the equivalent positions on the Talairach and Tournoux atlas [23]. The scaled images were then averaged to construct the MNI-250 brain template [66]. Then, another set of 55 MRIs was linearly automatically registered to the MNI-250 brain template. The 55 automatic and 250 manually registered brain images were averaged to create the MNI305 that has defined the MNI space [67]. The MNI152 template corresponds to 152 T1-weighted MRI linearly coregistered (nine-parameter affine transformation) to the MNI305. This new template exhibits better contrast and better definition of the top of the brain and the bottom of the cerebellum (25.02 ± 4.9, 86 males and 66 females) [68]. Another common template is the fsaverage atlas, based on 40 adults (10 each of young, middle-aged, and elderly adults, plus 10 Alzheimer’ s disease patients) [69]. The fsaverage is a dual template that references both volumetric and surface coordinates [64]. Recently, it has been common to use Nonlinear ICBM MNI (2009a, 2009b, and 2009c), which corresponds to the nonlinear registration of 152 MRI to MNI space [70].

### 2.6. Electrical Conductivity

Knowing the electrical conductivity values of the tissues that make up the human head is necessary to build reliable models that allow for greater accuracy in data analysis and experiment planning. A complete description of the electrical properties of the head’s tissues, particularly the skull and scalp, is given elsewhere [71]. The skull is highly resistive with respect to the surrounding tissues, which produces an electrical shielding effect between the areas outside and inside the skull. On the other hand, the conductivity of the scalp has a significant impact on tES since it is the first layer to be in contact with the electrodes. The conductivities of these two layers are usually modeled as homogeneous and isotropic, i.e., with a constant equivalent value and no preferential direction, although they can also be modeled as inhomogeneous [72] or anisotropic where the tangential conductivity differs from the radial [8]. The conductivity values assigned to each tissue are typically taken from the literature. New techniques, such as bounded electrical impedance tomography, allow minimally invasive, in vivo, and individual-specific conductivity estimation [73,74].

## 3. The Use of Computational Electric Field Modeling in tES

In this section, individualized-level knowledge of the electric field is reviewed, considering the importance of inter- and intra-individual effects (Section 3.1). Also, population-level knowledge of electric field distributions is reviewed, considering recent registration methods from the individual to brain template (Section 3.2). Then, studies describing the relationship between computationally estimated electric fields and measured physiological responses are reviewed in Section 3.3. Finally, strategies for optimizing tES montage are reviewed in Section 3.4. The studies identified in this review are based on a search strategy presented in Appendix A, developed for each section from Section 3.1, Section 3.2, Section 3.3 and Section 3.4.

### 3.1. Individual-Level Electric Field

The development of computational pipelines facilitates the automatic generation of individualized high-quality head models, allowing a systematic analysis of the anatomical factors in large data sets [16]. Individualized head models have identified the importance of brain tissue volume, thickness, and shape. Another factor of electric field variability is the cerebral spinal fluid thickness, which is also associated with the aging of the brain [19]. There have been efforts to describe the complexity of the brain in more detail. They include anisotropy of brain tissues [75] and emulation of small tissue compartments [76]. Earlier studies have also addressed the effects of current densities on the lesioned brain using simplified models and simulated brain lesions [77,78,79]. The following reviewed nine studies investigated the individualized electric fields on lesioned or non-neurotypical brain populations and summarized in Table 1.

Galletta et al. (2015) investigated cortical electric field patterns for five different bipolar tDCS montages in the head model of a post-stroke aphasia patient. The position of both electrodes is significant during tDCS clinical trials [80].

Minjoli et al. (2017) investigated the impact of changes produced in the spatial distribution of two head models with stroke lesions. Lower electric fields were observed by the increased proportion of the applied current shunted by a thicker CSF layer in the affected area [81].

Mahdavi et al. (2018) showed that a decreasing gray matter volume in three mild cognitively impaired head models reduced the magnitude of the current density in the brain compared to a young head model [82].

Lu et al. (2019) showed an increase in the scalp-to-cortex distance of the left primary motor cortex in dementia patients, which is markedly correlated with reduced tDCS-electric field compared to cognitively normal adults of comparable age [83].

Unal et al. (2020) used three head models with different degrees of local cortical atrophy. The difference in electric fields falls within the distribution of anatomically typical adults. Electric fields are not predicted simply by local morphological measures such as distance from the skull to the brain [84].

Indahlastari et al. (2021) showed that integrating white matter lesions into the models altered the current densities to 7% overall in white matter and 3% in gray matter outside lesion regions compared with non-lesioned brains. Changes in current density and total lesion volume were positively correlated [85].

Piastra et al. (2021) implemented a pipeline to automatically generate realistic and individualized volume conduction head models of chronic stroke patients. For large lesions, the authors found high differences in the electric field [86].

Jiang et al. (2022) showed that electric field distribution could be effectively predicted during tES in the lesioned brain of one epilepsy participant for the first time. The minimum correlation between measured via implanted stereo-electroencephalography electrodes and computed voltage distributions for different montages was significant [87].

Kalloch et al. (2022) showed that white matter lesions do not perturb the electric field globally and can thus be omitted when modeling subjects with a low-to-medium lesion load. However, the contribution of the white matter lesions significantly increased by ten and above in subjects exhibiting a high lesion load [88].

In summary, there has been a trend in computational analysis investigating tES-generated electric fields in non-neurotypical brains in the last five years, in contrast to previous studies focusing on neurotypical head models. Various studies have shown that lesioned or atrophied brain areas significantly affect the current distribution and intensity [80,81,82,83], depending on the lesion size and the target-to-lesion distances [84,88]. The development of advanced segmentation methods, which allow for the detailed incorporation of brain lesions [85,86] and recent validation of electric-field analysis in lesioned brains, allows the application of electric-field analysis in more challenging modeling situations [87]. These advancements indicate the potential to bring electric field analysis closer to clinical application in tES.

### 3.2. Population Level Electric Field

Properly comparing the electric field between subjects is difficult considering morphological variations (aging, brain atrophy, neurological dysfunction, and individual differences). Using brain templates as a standard space to register the electric field distribution from the individual brains allows us to better understand electric field trends in a population. Twelve studies considering a group-level approach are identified and summarized in Table 2.

Laakso et al. (2015) introduced a method to investigate the electric field in a standard brain space. Group-level electric fields concentrated near the primary motor cortex for a common electrode montage based on the 10/10 system despite subject-specific variations. The standard deviation was approximately 20% of the mean of the electric field on the target [18].

Laakso et al. (2016) used bipolar electrodes based on EEG landmarks to target hand motor and frontal areas. Electric fields could be controlled at the group level by adjusting the electrode location in the motor cortex. However, controlling the group-level electric field in the frontal area was more difficult, resulting in a larger variability of electric fields compared to the motor area. The authors also showed that not only do consistent group-level electric fields appear in the target area, but simultaneous significant fields also appear in other regions [24].

Csifcsák et al. (2018) found that there was no substantial difference between group-level electric fields between healthy and major depressive disorder (MDD) groups (19 healthy adults and 19 MDD). MDD-associated anatomical variations are not likely to influence current flow [89].

Gomez-Tames et al. (2019) showed that consistently significant fields in distinct functional networks of the cerebellum emerged despite inter-individual variations [25]. The hotspot value appears immediately under the active electrode with a negligible contribution of the return electrode location. The relative standard deviation was three times larger than that of the motor hand area [18].

Gomez-Tames et al. (2020) demonstrated that significant group-level hotspots appear in deep brain regions in contrast to the assumption that only cortical regions are consistently stimulated across subjects. Deep brain regions can be directly modulated by 70% of the cortical electric field strength [90].

Indahlastari et al. (2020) showed a negative correlation between the electric field and age in the DLPF and precentral gyrus using a customized template for the aging brain. The customized template produced a better representation of enlarged lateral ventricles, CSF content, and gaps between gyri curvature than the template based on young adults (ICBM-152) [91].

Rezaee et al. (2020) investigated the electric field strength in the cerebellum using MRI-averaged template models representing 18 different age groups (between 18 and 89 years old). The electric field decreased and dispersed to non-target cerebellar areas with aging due to cerebellar shrinkage and CSF volume increment [92].

Soleimani et al. (2021) investigated group-level electric fields on atlas-based parcellated areas. They considered modulation of global networks based on the high diffusion of the electric current in the brain in bipolar electrodes. The network-led approach showed a significant difference between montages for the left limbic system [93].

Antonenko et al. (2021) showed that the electric field distribution did not differ between young and older adult populations. However, both groups presented variability up to 25% of the field peaks. Also, they confirmed a robust inverse relationship between cortical electric field strength and extracranial and intracranial tissue volumes [94].

Suzuki et al. (2022) used group-level analysis to select the tACS montage for targeting the hand motor area. Electrodes were selected to maximize group-level electric field intensity on the putative brain area while reducing the inter-variability of the electric field [95].

Bhattacharjee et al. (2022) showed different electric field trends between genders across different age cohorts. The female group in the older age cohort had higher current densities than their male counterparts. No sex differences were observed in the middle-aged group. Males in the younger age group had a higher current density than females for montage targeting the parietal lobule [96].

Mizutani-Tiebel et al. (2022) compared three populations (major depressive disorder, schizophrenia, and healthy) for bifrontal montage. The group-level electric field strength in the prefrontal cortex was considerably lower for the major depressive disorder and schizophrenia patients compared to the healthy group, with no difference between the major depressive disorder and schizophrenia groups [97].

In summary, there is consensus that there is consistent delivery of electric fields in different brain functional areas based on common electrode positions to all subjects, even when dealing with diverse clinical patients ([18,24,25,90,92,93,94]). Adjusting the montage location to maximize the group-level electric field at the desired target should involve focusing the electric field in the target while minimizing its variability [95]. Furthermore, the notion that group-level electric field hotspots vary between different age groups and genders is supported in various studies ([91,92,96,97]). Conversely, small group-level electric field differences within the same age were observed between healthy and clinical population groups ([89,97]). It is noteworthy that the selection of a template may not substantially alter group-level electric fields, although customized templates tailored to specific features of a specific population may be beneficial ([24,91,93]).

**Table 2:** Summary of group-level electric field studies.

Study	Template	No. Subjects	Target	Montage	Notes
Laakso et al. (2015) [18]	Nonlinear MNI ICBM 2009a	24 healthy male subjects (38.63 ± 11.24)	Hand motor area	C3-Fp2 (35 cm^2^)	Mean electric field of 20%.
Laakso et al. (2016) [24].	・Custom averaged template (Yeo et al., 2010) ・Nonlinear MNI ICBM 2009a	62 healthy subjects (29.2 ± 11.2 years, 12 female)	Motor and frontal areas	16 bipolar montages (35 cm^2^)	Different templates do not produce significant differences for the group-level electric field in the target population.
Csifcsák et al. (2018) [89]	Fsaverage template using flatmaps (Pycortex)	・19 patients with major depressive disorder (33.52 ± 13.35 years) ・19 healthy adults (28.79 ± 10.86 years).	Prefrontal cortex	9 bipolar montages (5 × 7 or 5 × 5 cm^2^) and two HD (4 × 1)	MDD-associated anatomical variations are not likely to substantially influence current flow.
Gomez-Tames et al. (2019) [25]	・Nonlinear MNI ICBM 2009a	18 healthy males (43.4 ± 9.8 years)	Cerebellum (seven functional networks)	15 bipolar montages (5 × 5 cm^2^ and 2 × 2 cm^2^)	Systematic target emerges beneath the active electrode in group-level analysis. Standard deviations of the electric field up to 55% of the mean.
Gomez-Tames et al. (2020) [90]	・Nonlinear MNI ICBM 2009a	18 healthy males (43.4 ± 9.8 years)	Seven deep brain regions	7 bipolar montages (5 × 5 cm^2^)	Group-level hotspots appeared in deep brain regions (<70% of the cortical electric field).
Indahlastari et al. (2020) [91]	・Customized template (UFAB-587) ・Nonlinear ICBM-152	587 healthy patients (51 to 98 years, 262 males)	Dorsolateral prefrontal cortex	Two bipolar montages (F3–F4, M1–SO, size of 5 × 5 cm^2^)	Customized template improved the accuracy of tES current prediction in an older adult population.
Rezaee et al. (2020) [92]	Age-group MRI brain templates and SUIT template for cerebellum	18 age-groups of healthy adults (18 to 89 years)	Cerebellum (28 lobules)	Bipolar montage (one electrode 1 cm below and 3 cm lateral to Iz and second over the right buccinator muscle (5 × 5 cm^2^)	Averaged cerebellar shrinkage and increasing CSF content can lead to increased off-target stimulation.
Soleimani et al. (2021) [93]	Fsaverage template	60 males with methamphetamine use disorder (35.86 ± 8.47 years)	Network (Yeo7 Schaefer-400 atlas and Brainnectomate atlas)	In the frontal site, a 4 × 1 HD ring-centered electrode over F3	Group-level head models may be compared to a standard head model when high precision is not required.
Antonenko et al. (2021) [94]	Fsaverage template	・20 young adults (20–35 years) ・20 older adults (64–79 years)	・Middle frontal gyrus, ・Left precentral gyrus ・Left inferior parietal gyrus	Six bipolar montages of 19.6 cm^2^	Normalized spatial distribution of the electric field did not differ between young and older adult populations. Higher variability observed in young compared to older adults.
Suzuki et al. (2022) [95]	Nonlinear MNI ICBM 2009a	18 healthy males (43.4 ± 9.8 years)	Hand motor area	Seven bipolar montages of 3.24 cm^2^	First selection of montage based on maximizing group-level electric field intensity and reducing variability.
Bhattacharjee et al. (2022) [96]	Talairach template	250 healthy subjects divided in ・Three Male groups: 36.6 ± 8.4, 52.7 ± 18.6, and 74.2 ± 7.6 ・Three female groups: 34.9 ± 8.7, 53.07 ± 7.8, 74.08 ± 7.5	・Middle frontal gyrus, ・Left precentral gyrus	Two bipolar montages (CP5-CZ and F3-Fp2, size of 5 × 5 cm^2^)	Higher group-level electric currents were received by young males compared to young females at the (the opposite was observed for the old age group).
Mizutani-Tiebel et al. (2022) [97]	Conte69 surface template	・25 subjects with major depressive disorder (5.5 ± 11.28 years) ・25 subjects with schizophrenia (38.1 ± 10.46) ・25 healthy subjects (36.9 ± 13.71)	Prefrontal cortex	F3–F4 (4.5 × 6.5 cm^2^)	There are significant differences in the group-level electric field between clinical and non-clinical populations, but no significant differences between the two populations.

### 3.3. Correlations between Electric Field Calculations and Responses

Investigating the relationship between the tES response and spatial information of the generated electric field in the desired target(s) provides evidence of the feasibility of the application of computational electric field analysis to account for physiological responses. Twelve studies were identified that report on the correlation between the computation of the electric field in anatomical human head models and tES-generated response (summarized in Table 3).

Kim et al. (2014) showed one of the first attempts to establish a relationship between the computed current density at a specific target brain area and the behavioral outcomes. Significantly higher stimulation currents were delivered to the DLPFC in the responding group than in the non-respond group for the verbal working memory (WM) task [98].

Antonenko et al. (2019) investigated associations between electric fields and neurophysiological modulations induced by tDCS. Individual tDCS-induced GABA modulation was positively associated with the simulated electric fields. Individual tDCS-induced modulation of the sensorimotor network strength also correlates with the electric field [99].

Kasten et al. (2019) showed a significant multiple linear regression analysis relationship between alpha power increase (α-band) after receiving 20 min of tACS [100].

Laakso et al. (2019) showed that variability in the MEPs after anodal tDCS could be partly explained by the normal component of the electric field [101].

Jamil et al. (2019) investigated the correlation between predicted electric field strength and tDCS-induced CBF changes. For anodal-M1 tDCS, all current intensities show a positive correlation. The higher electric field in the cortex predicted a greater CBF increase relative to the sham group (1.5 and 2.0 mA showed a stronger association). For cathodal-M1 tDCS, a negative correlation was observed [102].

Abellaneda-Pérez et al. (2021) utilized two multifocal montages based on a measured fMRI pattern in thirty-one cognitively healthy older adults. The authors found that resting-state fMRI activity was topographically consistent with the calculated electric current density distribution. Also, specific individual tDCS-induced resting-state fMRI modulation had a significant negative correlation with the magnitude of the simulated electric current density estimates in the middle temporal gyrus [103].

Indahlastari 2021 et al. (2021) investigated the relationship between tDCS-generated electric current and functional connectivity changes. Significant correlations were found between functional connectivity (left DLFC and left VLPFC) and computed current densities in the left DLPFC. Sham stimulation showed no correlations between current density and functional conductivity [104].

Mezger et al. (2021) observed a trend of reductions in glutamate levels in participants who received large electric fields but not in the small electric field group during tDCS [105].

Zanto et al. (2021) investigated tACS effects on three groups: long exposure (6-Hz, 80 min total in three days), short exposure theta (6-Hz tACS, 16 min), and control group (1-Hz, 80 min). Only the long theta group discrimination performance change was correlated with a high electric field in the prefrontal cortex and peak theta frequency [106].

Nandi et al. (2022) showed that the averaged electric field is related to the anodal tDCS-induced GABA drop in the primary motor cortex. Temporal tDCS did not show a significant relationship [107].

Preisig et al. (2022) investigated gamma (40 Hz) tACS-induced changes in concurrent fMRI and auditory perceptual changes. The author used two high-density electrode configurations (in-phase and anti-phase) in addition to the sham condition. The authors found only a significant correlation between the electric field and interhemispheric connectivity in the anti-phase condition. They also found a statistical trend for the effect of electric field strength on tACS-induced BOLD signal changes in both hemispheres. The electric field had no direct influence on dichotic stimuli [108].

Yuan et al. (2023) examined the effect of anodal tDCS on functional connectivity in stroke survivors. The results showed that anodal tDCS increased functional connectivity within the ipsilesional sensorimotor network, and individual electric fields (normal component) predicted the changes in functional connectivity. No relationship was found with the sham condition [109].

In summary, recent works have agreed to find a significant association between various neurophysiological effects and computed electric fields in healthy young adults [99,100,102,103]. Recent evidence shows that this also applies to non-neurotypical brains and adult populations [85,109].

**Table 3:** Summary of the computed tES-generated electric fields and experimentally measured tES-generated responses.

**Study**	**tES Modality**	**Measurement**	**Physical Quantity**	**Relationship**	**Montage**	**Amplitude** **(mA)**	**Duration** **(min)**	**No. Participants**
Antonenko et al. (2019) [99]	Anodal Anodal Cathodic Anodal Cathodal	GABA GABA GABA SMN * SMN	E-Field Strength Normal Component E-Field Strength Normal Component E-Field Strength	・*r* = 0.53, *p* = 0.013 ・*r* = 0.47, *p* = 0.032 ・*r* = 0.45, *p* = 0.027 ・*r* = 0.53, *p* = 0.015 ・*r* = −0.49, *p* = 0.017	C3 (5 × 7 cm^2^)– SO (10 × 10 cm^2^)	1	15	24 young adults (25 years)
Kasten et al. (2019) [100]	tACS (α-band, 10 Hz)	EEG power increase	Electric field Strength	・*R*^2^ = 0.76 (positive), *p* < 0.001	Cz (7 × 5 cm^2^)–Oz (4 × 4 cm^2^)	1 (peak-to-peak)	20	40 young healthy adults (24.3 ± 3 years)
Laakso et al. (2019) [101]	Anodal tDCS	MEP	Normal Component	・*r* = −0.63, *p* = 0.0005	M1–SO (5 × 5 cm^2^)	1	20	28 healthy young adults (27 ± 6 years)
Jamil et al. (2019) [102]	Anodal tDCS	Cerebral perfusion change	Electric field Strength	・*r* = 0.295, *p* < 0.001	M1 (35 cm^2^)–SO (10 × 10 cm^2^)	1.5	90	29 young healthy adults (25.0 ± 4.4 years)
Abellaneda-Pérez et al. (2021) [103]	tDCS	Resting fMRI	Current Density	・*r* = −0.401, *p* = 0.0023	Multifocal montage (8 circular electrodes of 8 cm)	4 mA maximum (2 mA limit per electrode	25	31 older healthy adults (71.68 ± 2.5 years)
Indahlastari et al. (2021) [104]	Anodal tDCS	Functional connectivity	Current Density	・*R*^2^ = 0.523 (positive), *p* < 0.05	F3–F4 (5 × 7 cm^2^)	2	12	15 older healthy adults (71.8, 61–82 years)
Mezger et al. (2021) [105]	tDCS	Glutamate	Electric field	*-*	F3–F4 (5 × 7 cm^2^)	2	20	25 young adults (23.7 ± 2.0 years)
Zanto et al. (2021) [106]	tACS	Performance rate (NeuroRacer paradigm	Electric field	・*R*^2^ = 0.28, *p* = 0.017 * ・*R*^2^ = 0.34, *p* = 0.012 ** ・*R*^2^ = 0.45, *p* = 0.003 *** * post-tACS ** 1-day follow-up *** 1-month follow-up	F3–F4 (3.14 cm^2^)	2 mA peak-to-peak	26.67	60 healthy older adults (60 to 80 years)
Nandi et al. (2022) [107]	Anodal tDCS	GABA	Electric field Strength	・*R*^2^ = 0.46 (negative)	M1–SO (5 × 7 cm^2^)	1	10/20	24 young adults (23 years)
Preisig et al. (2022) [108]	tACS (α-band, 40 Hz)	EEG power increase	Electric field Strength	・*r* = −0.30, *p* = 0.13	Two high-density montages (Cp4 and Cp6). Inner electrode radius: 1.25 cm. External electrode: inner/external radius of 3.9/5.0 cm.	1.5 mA (peak-to-peak)	7	27 young healthy adults (21.9 ± 3.1 years)
Yuan et al. (2023) [109]	Anodal tDCS	resting fMRI	Normal Component	・*r* = 0.84, *p* < 0.001	C3/C4 and FP1/FP2 (5 × 5 cm^2^)	1	20	25 older stroke participants approximately (61 years)

SMN: sensorimotor network.

### 3.4. Montage Optimization

The computational modeling of the electric field allows the optimization of the current injection pattern, i.e., the locations and/or current injection intensities of each electrode, to deliver an electric field spatial distribution as close as possible to a desired one. The optimization varies according to the different desired characteristics of the resulting electric field distribution. For instance, one optimal pattern can maximize the electric field intensity in a specific region of interest (ROI) along a desired orientation, whereas another optimal pattern maximizes the focality, typically defined as some ratio between the ROI and no-ROI brain stimulation.

**Figure 3:**
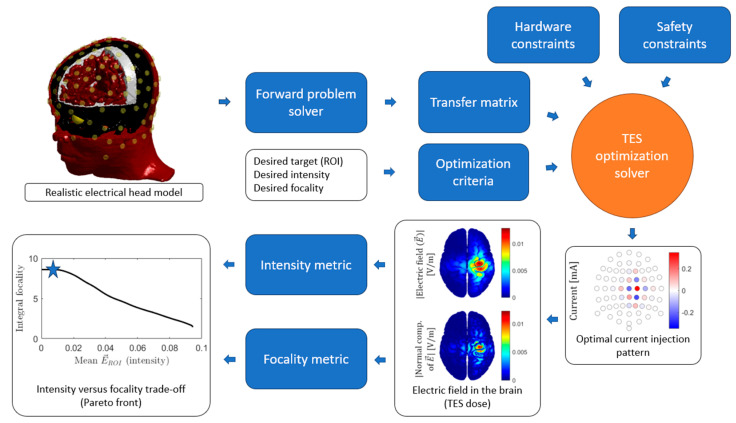
General tES optimization process. For an individualized head model, the forward problem is computed for every independent current injection pair to obtain the transfer matrix. The desired ROI is defined together with the optimization criteria, such as maximum focality of maximum intensity. The hardware (e.g., maximum number of electric current generators) and safety constraints (e.g., maximum current intensity per electrode) complete the specifications needed as inputs to the optimization solver. The result is a set of currents per electrode, i.e., an optimal current injection pattern. The forward problem can be computed for this pattern to obtain the electric field or current density map on the brain. From this map, the resulting intensity and focality can be computed, and these will represent a point (marked with a star for this example) in the intensity versus focality trade-off curve, also known as the Pareto front, that shows the performance of a set of optimal solutions.

Here, we consider only spatial optimizations based on the computational modeling of the electric field. In addition to spatial optimization, temporal characteristics of the electric field can be optimized, such as frequency or waveform, based on experimental trials or neuronal dynamic modeling, but temporal optimization is outside the scope of the current review. Figure 3 shows an overview of the optimization process. Twenty-four studies were identified in this section.

#### 3.4.1. Methodology

The simplest optimization problem is to maximize the electric field of the ROI along a predefined orientation given a total budget for the current injection (typically 1–4 mA). This is a convex optimization problem belonging to the class of linear programming problems and can be solved iteratively [28,110] or directly using the reciprocity principle [111]. An additional constraint may be the maximum current per electrode, solved iteratively [28,29] or using a closed form [111]. The next degree in complexity is adding a constraint on the electric field at the non-ROI, either on the sum of its energy [29,112] or on its peak [113]. This constraint can be posed as a linearly constrained quadratic problem [112] or as a quadratically constrained linear problem [113]. Depending on the value set on this constraint, a more or less focal solution is obtained [30]. Weighted least squares (WLS) optimization solutions were also proposed by minimizing the square of the difference between the resulting and the desired electric field maps [114]. It has been shown that these optimal solutions result in maximum focality patterns [30]. The norm of the current injection pattern can be added as another term to the WLS functional, resulting in solutions that distribute the injected electric current on more electrodes to reduce the skin sensation [115,116]. Another proposed constraint is to limit the angle between the desired and resulting electric field orientation in the ROI [29]. Moreover, forcing the solution to align with the desired electric field perfectly is possible using the Linearly Constrained Minimum Variance (LCMV) algorithm [28]. A more complex constraint is to limit the number of active electrodes, resulting in a combinatorial problem that can be solved using genetic [114] or branch and bound algorithms [29]. If no predefined orientation is assumed, the problem is not convex, but it can be solved using interior point algorithms [117,118]. The reviewed papers are summarized in Table 4.

Im et al. (2008) investigated the determination of optimal electrode positions for conventional two-electrode tDCS. The optimization problem they solved is equivalent to the problem of searching for two electrode locations that can generate maximal current toward a certain direction at the target brain area with a fixed current injection. Although they solved it using an iterative Evolutionary Strategy algorithm, their results appear to be consistent with the solutions to the same problem using the reciprocity principle [110].

Dmochowski et al. (2011) proposed multiple optimization schemes using a fixed multi-electrode array: WLS and LCMV, both with and without individual electrode current limits (“constrained” and “unconstrained”), and also directional maximization. Unconstrained LCMV and WLS were solved using closed formulas. Constrained LCMV and WLS and directional maximization were solved using Matlab convex programming tools. They found that WLS attains larger focality than LCMV at the expense of misaligned field direction at target. Also, intensity and focality can be substantially improved over conventional ad hoc approaches [28].

Park et al. (2011) proposed the maximization of the module of the electric field without imposing an a priori orientation. They solved the problem using the Nelder–Mead simplex method. The optimization was applied to a non-standard electrode array proposed by the authors. They found that the average current density values at the target areas were increased after the optimization [117].

Sadleir et al. (2012) also maximized the module of the electric field without imposing an a priori orientation and using an interior point algorithm, but the method did not guarantee that the solution attained was a global minimum. They concluded that when deep structures were targeted, it was not possible to avoid delivering current to peripheral cortical regions [118].

Ruffini et al. (2014) solved a constrained WLS problem, given a desired electric field map. Constraints were placed on the maximum current per electrode and the maximum total current. They used a genetic algorithm to solve the problem, including a limitation on the number of electrodes [114].

Guler et al. (2016) presented the directional maximization problem with an additional constraint in the non-target electric field energy to increase the focality. The problem was solved iteratively using convex programming methods. They concluded that increasing this additional boundary did not improve the objective function but increased the current density intensity [112].

Fernández-Corazza et al. (2016) proposed a straightforward solution for the directional maximization of the electric field problem based on the reciprocity principle. Basically, the optimal stimulation corresponded to the points in the scalp of maximum potential difference when a synthetic EEG dipole source was placed at the target. This approach could be used to consider the total- and per-electrode constraints [111].

Wagner et al. (2016) also included an additional constraint to the directional maximization problem for the non-target brain region instead of the energy of the peak of the electric field. They used an alternating direction method of multipliers (ADMM) algorithm to solve the problem. They found that optimization with this additional constraint improved the focality of the solutions [113].

Saturnino et al. (2019) proposed a large set of optimization problems (10 problems in total) that can be grouped into two families: quadratic problems that maintain the desired electric field at the target and maximize focality, and linear problems that maximize intensity. They included two additional constraints: one in the minimum angle between the resulting and the desired electric field and another in the number of active electrodes. They implemented their own convex optimization solvers and used the Branch and Bound algorithm to solve the combinatorial problem of limiting the number of electrodes [29]. Most optimization problems described in this work are included in the SimNIBS toolbox.

Fernández-Corazza et al. (2020) presented a unified framework linking the WLS and the reciprocity-based solutions to the constrained directional maximization iterative solutions. They found that the WLS solutions represent one end of the optimal solution set where the focality is maximal and the intensity minimal and that the reciprocity-based optimal solutions lie in the other end, where the focality is minimal and the intensity is maximal. Moreover, they show that changing the non-target constraint makes the transition between these two extremal solutions smooth, even for a discrete set of fixed electrodes [30].

Khan et al. (2022) presented a distributed constrained directional maximization approach, with the addition of a smooth constraint in the spatial distribution to reduce the side effects of skin-level sensations [115].

Galaz Prieto et al. (2022) presented a linear programming approach that performs L1-norm fitting and penalization of the current pattern (L1L1) to control the number of non-zero currents. They compared this approach with an LS approach (L2) using a smooth L1-norm constraint in the solution [116].

Wang et al. (2022) included a focality term for the directional maximization problem, which constituted an equivalent problem to [112], and solved it using standard convex optimization tools. They also analyzed the impact of conductivity uncertainty, showing that first the skull and second the scalp are the most relevant tissues [119].

#### 3.4.2. tTIS Optimization

tTIS can also be optimized, although it is a more complex and computationally intensive problem. A brute-force approach has been proposed using two pairs of electrodes [120]. Huang et al. (2020) made a major breakthrough by developing a directional electric field maximization algorithm for tTIS [31]. More recently, a package that solves the tTIS optimization problem for a set of optimal solutions was developed [121].

Rampersad et al. (2019) performed an exhaustive search with 88 electrode locations covering the entire head to optimize tTIS for target field strength and focality. They found that deep brain areas received field strengths similar to conventional tACS but with less stimulation in superficial regions. This suggests that tTIS can produce more focal fields and allows for better steerability than conventional tACS [120].

Huang et al. (2020) presented an optimization framework for tTIS using an array of electrodes and two frequencies. They found that maximal modulation intensity is achieved when tTIS equals conventional high-definition multi-electrode transcranial electrical stimulation (HD-tES) with a modulated current source. Once currents were optimized numerically to achieve optimal focal stimulation, they found that tTIS can be more focal than conventional HD-tES [31].

Wang et al. (2023) developed an evolutionary algorithm that maximizes the module of the electric field both for tES and for tTIS, generating a Pareto front (focality versus intensity trade-off) with a set of optimal solutions. In agreement with the previous work, they also found that optimized tTIS was more focal than optimized tES. They also highlighted the need for individualized stimulation protocols [121].

**Table 4:** Summary of tES optimization methods.

**Ref** **.**	**Study**	**Approach**	**Constrain** **ts**	**Problem Type**	**Algorithm/** **Function**	**Note**
[110]	Im et al., 2008	Directional Maximization	Max total current	Convex	Evolutionary Strategy	
[28]	Dmochowski et al., 2011, Problem 1 (P1)	WLS	・None	Convex	Closed formula	
	Dmochowski et al., 2011, (P2)	Constrained WLS	・Max total current ・Max per electrode current	Convex	Matlab disciplined convex programming	
	Dmochowski et al., 2011, (P3)	Minimize energy with the fixed field at the target (LCMV)	・Fixed field at target	Convex	Closed formula	
	Dmochowski et al., 2011, (P4)	Minimize energy with the fixed field at the target (LCMV)	・Fixed field at target ・Max total current ・Max per electrode current	Convex	Matlab disciplined convex programming	
	Dmochowski et al., 2011, (P5)	Directional Maximization	・Max total current	Convex	Matlab disciplined convex programming	Equal to [110] but solved differently.
[117]	Park et al., 2011	Module Maximization	・Max total potential	Non-convex	Nelder–Mead	
[118]	Sadleir et al., 2012	Module Maximization	・Max total current ・Max non-target intensity (focality) ・Min target/non-target ratio (focality)	Non-convex	Interior-point (Matlab fmincon)	Similar to [117]
[114]	Ruffini et al., 2014	Constrained WLS	・Max total current ・Max per electrode current ・Max number of active electrodes	Combinatorial	Genetic Algorithm	
[112]	Guler et al., 2016	Directional Maximization	・Max total current ・Max per electrode current ・Max non-target energy (focality)	Convex	CVX Matlab package [122]	Evolution of [28] (P5) (more constraints)
[111]	Fernández-Corazza et al., 2016	Directional Maximization	・Max total current ・Max per electrode current	Convex	Closed formula	Evolution of [28] (P5) (more constraints) Included in [112] (less constraints)
[113]	Wagner et al., 2016	Directional Maximization	・Max total current ・Max per electrode current ・Max non-target intensity (focality)	Convex	Alternating direction method of multipliers (ADMM)	Small difference with [112]
[123]	Saturnino et al., 2019 P1	Minimize energy	・Fixed normal component at Target	Convex	Active-set (Python)	Similar to [28] (P4) but fixing the normal component instead of the three components of the electric field.
	Saturnino et al., 2019 P2	Directional Maximization	・Max total current ・Max per electrode current	Convex	Active-set (Python)	Equal to [111]
	Saturnino et al., 2019 P3	Minimize energy	・Fixed normal component at Target ・Max total current ・Max per electrode current	Convex	Active-set (Python)	Similar to [28] (P4) but fixing the normal component instead of the three components of the electric field.
	Saturnino et al., 2019 P4	Minimize energy	・Fixed normal component at Target ・Max total current ・Max per electrode current ・Max number of active electrodes	Combinatorial	Branch and bound	
	Saturnino et al., 2019 P5	Directional maximization	・Max total current ・Max per electrode current ・Min angle	Convex	Active-set (Python)	The angle restriction depends on the solution, which makes it iterative (no examples shown)
	Saturnino et al., 2019 P6	Minimize energy	・Fixed normal component at Target ・Max total current ・Max per electrode current ・Min angle	Convex	Active-set (Python)	
	Saturnino et al., 2019 P7	Minimize energy	・Fixed normal component at Target ・Max total current ・Max per electrode current ・Min angle ・Max number of active electrodes	Combinatorial	Branch and bound	
	Saturnino et al., 2019 P8	Minimize energy	・Fixed normal component at many targets ・Max total current ・Max per electrode current	Convex	Active-set (Python)	
	Saturnino et al., 2019 P9	Directional maximization for many targets	・Max normal component at the targets ・Max total current ・Max per electrode current	Convex	Active-set (Python)	
	Saturnino et al., 2019 P10	Minimize energy	・Fixed normal component at many targets ・Max total current ・Max per electrode current ・Max number of active electrodes	Combinatorial	Branch and Bound	
[30]	Fernandez Corazza et al., 2020	Directional maximization	・Max total current ・Max per electrode current ・Max non-target energy or intensity (focality)	Convex	Matlab CVX and closed-form	Links [112] with [28] (P1), [112] with [111], and [113] with [111].
[115]	Kahn et al., 2022	Directional maximization	・Max total current ・Max per electrode current ・l1 norm in the solution	Convex	[not stated]	
[116]	Galaz Prieto et al., 2022	Weighted L1 norm	・Max total current ・l1 norm in the solution	Convex	Matlab CVX	
		WLS	・Max total current ・l1 norm in the solution	Convex	Matlab CVX	
		WLS with Tikhonov regularization	・Max total current ・Total energy (focality) ・l2 norm in the solution	Convex	Matlab CVX	
		Weighted L1 norm	・Max total current ・l1 norm in the solution ・Max number of active electrodes	Convex	Matlab CVX	The max number of active electrodes constraint is forced (not optimal) in a second step
		WLS	Same as above	Convex	Matlab CVX	Same as above
		WLS with Tikhonov regularization	・Max total current ・Total energy (focality) ・l2 norm in the solution ・Max number of active electrodes	Convex	Matlab CVX	Same as above
[121]	Wang et al., 2022	Directional maximization	・Total energy (focality) ・Max total current ・Max per electrode current	Convex	Matlab CVX	Equivalent to [112]

#### 3.4.3. Applications of These Methodologies to Simulated Experiments

There are some studies that analyze the performance of some of the previously described methodologies applied to a particular context based on simulations. The electric field directional maximization approach was applied to stroke rehabilitation [124,125] and parietal stimulation [126]. The LS approach was applied to simulate optimal cerebellar stimulation [26]. Optimized tTIS was used to stimulate the hippocampus based on an exhaustive search [127].

Dmochowski et al. (2013) applied the directional maximization approach to realistic head models of eight stroke patients with chronic aphasia. The peri-lesional cortical areas with the highest BOLD activation during the task were deemed the targets for treatment. They demonstrated that electric field strengths in the targeted cortex could be substantially increased over the conventional approach [124].

Rezaee et al. (2019) applied an LS approach to optimize stimulation in two cerebellar targets, comparing it with a 4 × 1 standard montage. They performed simulations on an individual head model and an atlas head model. They concluded that optimized montages can facilitate the rehabilitation of impaired standing balance [26].

Radecke et al. (2020) applied the distributed constrained directional maximization and the ADMM approaches to realistic head models of 21 healthy subjects, targeting the parietal cortex in three different directions. Their results supported using individual targeting to enhance the efficacy of tES and elucidate its underlying mechanisms [126].

Lee et al. (2020) optimized scalp electrode configurations and injection currents to deliver maximum tTIS stimulation currents to the head of the right hippocampus, considering the real anatomical head structures of three individuals. They obtained the optimal two pairs of this stimulation by carrying out an exhaustive search [127].

Cruijsen et al. (2022) applied the directional maximization approach to realistic head models of 21 stroke patients and 10 healthy subjects. They set the motor hand knob as the stimulation target. They found that optimized electrode configurations increased the electrical field strength in the anatomical and functional target for subjects with stroke but did not reach the same levels as those in healthy subjects [125].

#### 3.4.4. Applications of These Methodologies to Real-Subject Experiments

There are a limited number of studies that applied some of the optimal methods to experiments with real subjects. Reciprocity-based optimal stimulation patterns were applied to twelve healthy subjects, seven epilepsy patients, and twenty healthy subjects in different studies [40,128,129].

Luu et al. (2016) applied the reciprocity principle to stimulate the M1 motor region on 12 healthy subjects with tDCS, resulting in the selection of 8 sources and 8 sinks from a 256 EEG sensor net. The results showed significant long-term depression (LTD) of the motor evoked potential as a result of the cathodal pulses (current flows outward from the cerebral cortex) in contrast to the placebo control, with a smaller but similar LTD effect for anodal pulses [40].

Holmes et al. (2019) examined the effects of slow-pulsed transcranial electrical stimulation (tES) in suppressing epileptiform discharges in seven adults with refractory epilepsy. Interictal spikes were localized, and tES targeted the cortical source of each subject’s principal spike population. They found that optimally targeted spikes were suppressed more than non-targeted spikes [128].

Klírová et al. (2021) optimally stimulated the medial prefrontal cortex to indirectly stimulate the anterior cingulate cortex. The subjects performed visual and cognitive tests before and after sham, as well as optimal and non-individualized stimulation. Compared with non-individualized stimulation, the individualized optimal stimulation significantly increased the number of interference words and the interference score in the Stroop test [129].

#### 3.4.5. Optimization Summary

In summary, a wide range of optimal problems and their solutions have been proposed for optimizing the spatial distribution of tES and tTIS. They were validated by means of realistic electric field modeling simulations and some real experiments in humans. The electric field can be maximized along a predefined orientation, which constitutes a convex optimization problem, or by its module, which constitutes a non-convex problem requiring more complex algorithms. Some solutions can be found with closed formulations, but most are found by iterative solvers. Besides the maximum total current and maximum per electrode safety constraints as well as Kirchoff’s rule, other novel constraints can be added to the problem, such as a limited number of active sources, an angular tolerance between the desired and the resulting electric field, or an L1 constraint to spatially smooth the solutions. The non-target constraint, either to the electric field energy or peak, is relevant for defining the optimal point within the intensity versus focality trade-off.

Regarding the different optimization approaches, the more complex they are, the larger their computational cost. Closed formula solutions, such as LS, LCMV, or solutions based on the reciprocity theorem, can be considered almost instantaneous, as they can be solved in less than a second [28,111]. Convex optimization problems with non-combinatorial constraints and up to 256 electrodes can be solved in a few seconds [112]. Convex optimization problems that impose restrictions on the number of electrodes solved using the Branch and Bound method can be solved in minutes [29]. tTIS optimization using the fminimax Matlab function is much slower, and each solution takes approximately 1–2 h on a standard PC [31].

In the case of clinical populations, the same optimization methods are valid if the disease is reflected in the anatomical model. For instance, a reciprocity-based optimal stimulation pattern is one candidate, such as in the case of epilepsy. This is because, in most cases, epilepsy foci are located in deep brain regions such as the hippocampus or the temporal lobe, and reciprocity-based solutions deliver the maximum intensity to the target for a given current injection budget. Applications that require more shallow stimulation, for instance, of the motor cortex for treating dementia or motor-related disorders, could benefit from more focal algorithms. However, the number of studies is still limited to investigate whether there are preferable optimization methods according to the disease.

## 4. Final Overview

This review presents new trends in the computational modeling and analysis of the spatial distribution of the electric field and optimization for tES. Here, we summarize and discuss the most relevant aspects and conclusions of this review as well as future perspectives in the field.

### 4.1. Application of Electric Field Analysis in Clinical Practice

The modeling of the spatial distribution of the electric field for transcranial electrical stimulation (tES) has advanced to a stage where diverse computational methods, implemented through various pipelines, can efficiently estimate the electric field in individual anatomical models. Computational analysis of electric fields has significantly contributed to understanding spatial-dependent characteristics of the electric field at individual- and population levels. Simulation results indicate that individually optimized current injection patterns should be preferred versus standard non-optimal ones. Additionally, electric field computational modeling can help account for inconsistent effects across studies by reducing variability in the electric field intensity, which is not possible by using the same montage and amount of current injection for all participants. There is a consensus that it is possible to consistently deliver electric fields to specific brain structures, even when dealing with diverse clinical patients.

The most recent studies have expanded beyond those limited to healthy individuals to include non-neurotypical brains and big data due to available MRI datasets. It is now possible to incorporate lesions that affect the spatial distribution of the electric field through advanced segmentation methods [46,47].

It is important to note that the computational model serves as a proxy for the real electric field, and ensuring its predictive accuracy is crucial for precise tES experimental design and analysis. Electric field analysis relies on individual anatomical features affected by the quality and fidelity of the digital representation of segmented tissues. The conclusion is that there is good evidence of the reliability of the analysis despite these limitations. First, different efforts show good agreement between independent numerical implementations for estimating the tES-generated electric field [130,131,132]. Second, various studies provide confidence in the numerical analysis based on agreement with real-world in vivo measurements of the electric field distribution in the brain [87,133,134]. Third, this review shows that computational models have also revealed a significant correlation between the computed electric field and the stimulation responses.

### 4.2. Electric Field Optimization

The computational analysis of the electric field has significantly contributed to enhancing the distribution of the electric field on specific targets through improved multi-electrode current injection patterns based on optimization techniques. The simulation results clearly show that individually optimized patterns should be preferred versus standard non-optimal current injection patterns. tTIS simulations show that it can be strongly improved with optimized stimulation patterns, even more than optimized tES. However, tTIS optimization is much more computationally intensive than tES optimization. Preliminary experimental results comparing optimal versus non-optimal patterns support the hypothesis that optimization methods significantly enhance the efficacy of tES and tTIS, although more experimental studies are still needed. In addition, the group-level approach is a complementary approach to individualized optimization with the aim of finding the optimal common montage to a population. It can maximize focality and minimize inter-variability of the electric field within a population. In addition, it provides advantages of electrode placement based on easy scalp landmarks or when the MRI data acquisition to construct the individual digital head model is not available for individualized electric field analysis. It is pending to quantify how well group-level-based optimization approximates individual modeling.

While some optimization methods aim to maximize the module of the electric field at the target, the maximization of a directional component of the electric field has been more extensively developed. This is due to two main reasons: first, the directional problem is simpler to solve, and second, it is assumed that the direction of the stimulation is highly relevant. The cortex is organized in columns of pyramidal (likely excitatory) neurons oriented perpendicularly to the cortical surface, which is the selected orientation in most studies. However, there are also interneurons (mostly inhibitory) that are aligned parallel to the cortical surface. Thus, which orientation should be selected for the target remains an open question. In any case, the directional optimization algorithms can optimize stimulation at any desired orientation. Notably, it is important to determine if any difference between tDCS and tACS effects is sensitive to the amount of electric field, considering that each method operates through distinct mechanisms.

### 4.3. Limitations

Despite the encouraging results presented in this review, it is still important to revisit various limitations of electric field modeling. For example, the conductivity of each tissue is commonly set at the same default value for all individuals. Some previous studies have indicated that age-related calcification changes can lead to a significant conductivity shift in the skull [71,135]. This alteration may impact the intensity level and, to a lesser extent, the spatial distribution [136]. Scalp conductivity is also thought to highly influence the modeling accuracy as it is the tissue in contact with the electrodes. Another example is mesh quality, which can derive significant imprecisions in the numerical solvers of the forward problem. For instance, meshing thin layers such as the CSF or the skull should not have one element that touches both the internal and external boundaries of these tissues [137]. The accuracy of the electrode placement is another source of modeling errors. Here, the same efforts performed to accurately determine them in EEG based on magnetic sensors or photogrammetry should also hold for tES. Overall, simulations showing the impact of uncertainties in the models, such as electrode locations, contact impedances, tissue conductivity values, segmentation errors, or mesh densities, are also required to analyze the robustness of the different optimization approaches in practical scenarios. Moreover, these uncertainties are expected to affect the accuracy of tTIS more than tES because two interfering fields must be spatially aligned in tTIS. Despite these limitations, it is important to mention that electric field analysis methods have been able to reproduce in vivo measurements of intracranial currents [87,134,138]. Nevertheless, further detailed experimental validation is needed to enhance the accuracy of electric field computation and improve parameter fitting, particularly when considering the impact of the model assumptions (e.g., conductivity and number of represented tissues) and uncertainties (e.g., mesh quality and electrode location).

Note that this review covers the spatial distribution of the electric field, which does not account for the impact of the specific temporal dynamics of the electric field interaction with neural modulation. Indeed, intensity- and duration-dependent effects on tDCS have been observed. This may explain the disagreement regarding the relationships between current intensity and induced after-effects of tDCS in monotonic or non-monotonic manners [37,139], which need to be clarified to improve the understanding of the involvement of a specific brain area in the examined clinical or behavioral outcomes [140,141].

### 4.4. Future Directions and Challenges

More tES and tTIS experimental studies using optimization techniques are needed to validate the simulation findings in real cases. One limitation is the lack of versatile hardware. Next-generation tES hardware, either prototypical or commercial, should have compatibility with multi-electrode caps, several independent and programmable current generators, and a controlled switching matrix to connect the generators to any electrode if the generators are less than the total number of electrodes. To allow the application of optimized tTIS patterns, the generators should support the generation of at least two higher frequencies (in the order of kHz). A system with these characteristics will facilitate more experimental studies using optimal current injection patterns. User-friendly software is also needed to make these tools accessible to experimental researchers and, subsequently, to clinical applications. This software should first have a simple, ideally a one-click button, model generator from the subject-specific images: structural T1 and, if available, T2 and/or CT. In this direction, SIMNIBS software has greatly contributed with the ‘charm’ function. Electrodes should be automatically placed in the model based on magnetic sensor or photogrammetry methods, where the user only needs to manually define some fiducial points in the model, although there are algorithms that automatically detect some of these points. The electrical conductivities should be introduced by the user, with literature values by default. The electromagnetic field modeling should be performed in the backend and be transparent for the user. Finally, the software should allow the user to easily select a desired target on the MRI and set the parameters of the stimulation, such as maximum total current, maximum current per electrode, and/or maximum number of independent current generators available in the hardware. After the simulation is run, the software should show the final user the optimal patterns and the resulting electric fields (intensity and direction) on top of the structural MRI. We believe that a slider allowing the user to select a different solution from the intensity–focality curve should be useful in practice. To enable the user to navigate along these solutions quickly, they should be precomputed in advance, for instance, for ten evenly distributed points of the curve, including the two extrema.

Regarding practical implications, the implementation of individualized computational optimization of tES may increase the cost (namely, acquisition of high-resolution structural MRI, specialized software for montage optimization, navigation equipment for electrode positioning, and user training), which may lose competitive advantages of tES, which are inexpensive and easy to apply. The benefits of individualized tES optimization have to outweigh the costs in future studies. One alternative is montage optimization based on group-level analysis of a representative population, which may continue to make tES as inexpensive and easy to apply as it does not require MRI acquisition and navigation equipment for electrode positioning. Nevertheless, how suboptimal group-level optimization compares with individual-level optimization requires investigation in future studies.

All the optimization methods included in this review can also be used to stimulate the brain using intracranial electrodes, either with electrocorticography grids or with deep stereo-encephalography electrodes. For instance, these intracranial electrodes are implanted in patients with epilepsy or Parkinson’s disease. Some studies have already used tES optimization methods to improve intracranial brain stimulation [142,143]. Electric field modeling can also improve closed-loop stimulation, which is the stimulation that is somehow triggered by an EEG or another biomarker. Moreover, multisite tACS targeting [17] is expected to be used to modulate functional brain networks.

In conclusion, current computational pipelines have reached a degree of maturity to obtain enough knowledge of the intracranial electric field at individual and group levels. Combining electric field simulations with advanced optimization techniques allows a high degree of control to deliver the electric field with the required focality and direction. Although there is evidence of the relationship between tES-generated intracranial electric field quantity and responses, more efforts are required to investigate the physiological effects of optimized and non-optimized protocols. Moreover, extending the current electric field analysis to include temporal effects by integrating biophysical dynamic neural modeling is important. At the same time, we provide suggestions for future works to bring tES closer to clinical practice based on the needs that come from the state-of-the-art works covered in this review.

## Figures and Tables

**Table 1:** Summary of tES computational studies on lesioned brains.

Study	Lesion	Affected Tissue Conductivity (S/m)	Montage (Area cm^2^)	Subjects (Years)
Galletta et al. (2015) [80]	Stroke (frontal area)	1.65	Five montages: CP5/F5/CP6/F6-SO, F2–F3 (5 × 7)	One adult male (N.A)
Minjoli et al. (2017) [81]	Stroke	N.A	Near cortical lesion (7)	Two-stroke adults (36 and 44) One healthy control (adults)
Mahdavi et al. (2018) [82]	Mild cognitive impairment	N.A	Two montages: T3/F3-SO (7 × 5)	Healthy youth (24), healthy elder (78), and mild cognitively impaired elder (78)
Lu et al. (2019) [83]	Dementia	N.A	C3–C4 (5 × 5)	164 cognitively normal adults (40 young age: 29.4 ± 4.0, 65 middle age: 50.2 ± 5.4, and 62 old age: 75.7 ± 8.1), and 43 dementia patients (76 ± 6.8)
Unal et al. (2020) [84]	Local cortical atrophy variant of primary progressive	Same as the surrounding tissue’s conductivity	-F7-right cheek (5 × 5) −4 × 1 high-definition centered over left IFG * area	Four adults (69, 59, and 71)
Indahlastari et al. (2021) [85]	Lesion volumes based on training data from multiple sclerosis patients	1.65	F3–F4 (5 × 7)	130 adults (71, 65–85 range)
Piastra et al. (2021) [86]	Stroke	0.126 to 1.654	C3-Fp2, C4-Fp1 (Area N.A.)	16 adults (N.A)
Jiang et al. (2022) [87]	Two congenital malacia foci	0.8	T7-Fp2, T7–T8 (3.14)	1 young adult (21)
Kalloch et al. (2022) [88]	White matter (divided into four Fazekas scores)	1 (beta distribution)	Oz-Fpz (25)	88 old adults (70.8 ± 4 years)

* IFG: left inferior frontal gyrus.

## Data Availability

No new data were created for this review.

## Appendix A

A search strategy was developed to retrieve papers for each subsection of Section 3.1, Section 3.2, Section 3.3 and Section 3.4. The search database was Web of Science, covering the period from 1990 to 01.05.2023. The papers retrieved were screened to assess soundness based on the title and abstract (“Relevant”). The full contents of the papers that passed were revised to discard or accept (“Included in analysis”). The Google Scholar engine was used to identify studies from 2023 that have not yet been indexed in the Web of Science. The details of the search strategy are presented in Table A1.

**Table A1:** Summary of search strategy results.

**3.1. Individual Level Electric Field**							
Search data	TS = (“tES” OR “tDCS” OR “tACS” OR “transcranial electri* stimulation” OR “transcranial direct current stimulation” OR “Transcranial Alternating Current Stimulation”) AND TS = (“electric field” OR “current density”) AND TS = (Comput* OR Model* OR Simulation$) AND TS = (Individual$ OR Subject$ OR Human$ OR Head$) AND TS = ((Brain$ or tissue$ or cereb*) and (atrophy* or lesion$)) NOT TS = (Animal$)						
Identified from database	23	Excluded (not relevant)	7	Relevant	16	Identified from other sources	0
Included in analysis	9						
**3.2. Population Level Electric Field**							
Search data	TS = (“tES” OR “tDCS” OR “tACS” OR “transcranial electri* stimulation” OR “transcranial direct current stimulation” OR “Transcranial Alternating Current Stimulation”) AND TS = (“electric field” OR “current density”) AND TS = (Comput∗ OR Model∗ OR Simulation$) AND TS = (Individual$ OR Subject$ OR Human$ OR Head$) AND TS = (Group NEAR/5 level OR Group NEAR/5 Simulat* OR Standard OR Template$ OR Atlas OR Warp* OR MNI OR Talairach) NOT TS = (Animal$)						
Identified from database	48	Excluded (not relevant)	29	Relevant	19	Identified from other sources	2
Included in analysis	12						
**3.3. Correlations between Electric Field Calculations and Responses**							
**Search data**	TS = (“tES” OR “tDCS” OR “tACS” OR “transcranial electri* stimulation” OR “transcranial direct current stimulation” OR “Transcranial Alternating Current Stimulation”) AND TS = (“electric field” OR “current density”) AND TS = (Comput∗ OR Model∗ OR Simulation$) AND TS = (Individual$ OR Subject$ OR Human$ OR Head$) AND TS = (Individual* OR Personal*) NOT TS = (Animal$)						
Identified from database	110	Excluded (not relevant)	69	Relevant	41	Identified from other sources	0
Included in analysis	12						
**3.4. Montage Optimization**							
**Search data**	TS = (“tES” OR “tDCS” OR “tACS” OR “transcranial electri* stimulation” OR “transcranial direct current stimulation” OR “Transcranial Alternating Current Stimulation”) AND TS = (“electric field$” OR “current densit*” OR “current flow field$”) AND TS = (Comput∗ OR Model∗ OR Simulation$) AND TS = (Individual$ OR Subject$ OR Human$ OR Head$) AND TS = (optimi*) NOT TS = (Animal$)						
Identified from database	79	Excluded (not relevant)	44	Relevant	35	Identified from other sources	5
Included in analysis	24						
